# Which Tissue Should Be Removed in Upper Blepharoplasty? Analysis and Evaluation of Satisfaction

**Published:** 2017-09

**Authors:** Ali A. Saalabian, Paul Liebmann, Maria Deutinger

**Affiliations:** Department of Plastic, Reconstructive and Aesthetic Surgery, Krankenanstalt Rudolfstiftung, Wien, Austria

**Keywords:** Blepharoplasty, Satisfaction, Complication, Rejuvenation

## Abstract

**BACKGROUND:**

Due to various options for tissue resection and preoperative markings, many different reports on aesthetics and patient’s satisfaction exist. To assess differences among tissue resections and risk factors, we herein analyzed satisfaction levels of patients that underwent upper blepharoplasty.

**METHODS:**

A retrospective analysis during the period from January 2006 to June 2013 was conducted by reviewing patient’s electronic medical files. All patients underwent medically indicated upper blepharoplasty at our department. We classified patients relating to resected tissues; hence the categories created were skin, skin/muscle, skin/muscle/fat and skin/fat. Furthermore, an evaluation of risk factors according to the patient’s number of present medical preconditions ranging from 0 (none) to 4 was performed. Data collection was conducted by reviewing patient’s electronic medical files. Moreover, a questionnaire concerning patient’s satisfaction was forwarded.

**RESULTS:**

No significant differences in patient’s satisfaction and complication rates comparing the different groups of tissue resection were noted. However, we found a significantly higher complication rate at a presence of 2 risk factors. In addition, a significantly worse scar outcome and longer recovery periods in patients with 4 risk factors were observed.

**CONCLUSION:**

The extent of tissue resection has no statistically quantifiable effect on patient’s satisfaction ratings and complications. For this reason, we believe cautious resection of muscle and fat is only indicated if pathologies are present. Moreover, patients with 2 risk factors or more shall be rigorously evaluated preoperatively to avoid complicating events.

An abbreviated form of this manuscript was presented at the conjoint 52^nd^ and 45^th^ annual meeting of the Austrian and German Society of Plastic, Aesthetic and Reconstructive Surgery, September 11^th^-13^th^ 2014 in Munich, Germany.

## INTRODUCTION

**Fig. 1:**
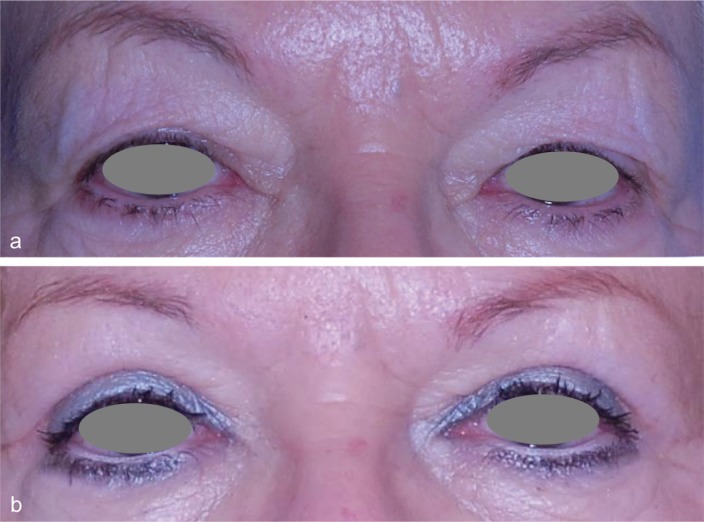
a) Pre- and b) postoperative view of a representative patient that underwent upper blepharoplasty

The eyes and the periorbital aesthetic subunit are integral part of a young facial appearance. Fullness of specifically the lateral aspect of the upper lid is characteristic for the youthful brow/upper eyelid complex,^1^ hence, restoration of depleted facial volume is key in periorbital rejuvenation (Figure 1).^2^^,^^3^ Due to lifelong exposure to ultraviolet radiation and the constant use of the mimic musculature, the face and the periorbital region in particular are therefore among the first areas to indicate aging processes. To nobody’s surprise upper lid blepharoplasty is among plastic surgery’s most common procedures in the US and ranks 3^rd^ in surveys.^4^^,^^5^

With the increasing popularity, questions referring to patient’s satisfaction, operative techniques, complication rates and risk factors have been raised: What are the effects of risk factors on complication rates, patient’s satisfaction-, scar- and aesthetic ratings as well as recovery periods? What influence has the type of resected tissue on these parameters? What types of tissue resections are most commonly used? To answer these questions, we herein present our experience with patients presenting for functional blepharoplasty concerning aesthetics and potential risk factors. In almost all patients the indication for surgery was restriction of visual field.

Assessment of the brow and forehead is fundamental to achieve good overall results. In aesthetic periorbital rejuvenation, it is essential to restore eyebrow position, symmetry and stability for integral treatment of blepharochalasis, if brow ptosis or rhytides of the forehead are present. However, the importance of simultaneous treatment of the brow and forehead and the intricacies in aesthetic functional eyelid surgery have been addressed extensively and will not be covered here as this would go beyond the scope of this article.^6^^-^^8^

Various images of a naturally appealing brow exist nowadays depending on ethnicity und cultural differences. However, generally accepted ideal relationships between the brow-eye-nose complex are described. The brow starts medially just at the supraorbital rim in a line drawn perpendicular to the alar base and ends laterally marked by an oblique line that connects the alar base with the lateral canthus. The highest point of the brow lies at the transition of the middle and lateral third just at an extended line through the lateral corneoscleral limbus. In men, the brow is located just at or slightly above the supraorbital rim. In women, the brow is situated 0.5 cm to 1 cm above the rim.^9^

An important anatomical landmark in surgery of the upper lid is the supratarsal crease. It can be found as an arched fold about 8 to 10 mm above the upper palpebral margin and is formed by the insertion of the aponeurosis of the levator palpebrae superioris, the orbital septum and the fascia of the orbicularis oculi into the dermis. In the aged the supratarsal fold tends to degenerate, which can cause ascending of the crease, lid ptosis and skin laxity of the upper lid.^9^

Notable differences in the eyelids of Asians compared to those of Caucasians are the more distal fusion of the orbital septum with the levator aponeurosis and the resulting descending of preaponeurotic fat.^10^ The orbital fat can then be further classified into a nasal medial (orbital) and a temporal lateral (preaponeurotic) compartment. Both can be found posterior to the orbital septum separated from each other by the superior oblique muscle.^9^

The fat in the medial compartment generally has a brighter coloring than the fat in the lateral compartment. The lacrimal gland, which in case of protrusion can cause impairment of visual field, can be found in the temporal compartment. Other structures found in the nasal compartment are the medial palpebral artery and the infratrochlear nerve.^11^ Finally, in some patients, most notably in the Asian population, existing preseptal retroorbicularis fat can lead to lid fullness and defacement of the supratarsal fold.^10^ To assess differences among tissue resections and risk factors, we herein analyzed satisfaction levels of patients that underwent upper blepharoplasty.

## MATERIALS AND METHODS

Electronic medical files of patients that received medically indicated upper lid blepharoplasties at our institution in the time period from January 2006 to June 2013 were retrospectively reviewed to identify presumed risk factors, to evaluate patient’s level of satisfaction regarding surgery’s outcome and to analyze tissue components resected in blepharoplasty. Seven different surgeons have been involved in performing blepharoplasty in the given period; hence slightly different surgical techniques were used. Additionally, all patients received a self-designed standardized questionnaire involving ten questions per mail to return to our address by using prepaid and pre-addressed envelopes. 

To maximize participation, to complement questions and resolve questionnaire-associated uncertainties, patients have also been contacted by phone. Overall, 387 patients (56.6%) agreed to participate in this study and were evaluated entirely, either by returned questionnaires by mail or per interrogation on phone. All procedures performed were in accordance with the ethical standards of the institutional research committee and with the 1964 Helsinki declaration and its later amendments.

The following parameters have been obtained by reviewing the patient’s electronic health records prior to our interrogation: (i) diagnosis; (ii) resected tissue; (iii) follow up; (iv) age; (v) sex; (vi) body mass index (BMI); (vii) nicotine consumption; (viii) presence of relevant conditions in past medical history; and (ix) complications. Following complications occurred: asymmetry, asymmetry with skin excess, epithelial cyst, long lasting swelling (>6 weeks), skin excess and wound dehiscence.

The survey involved ten questions that covered the issues patient’s assessment of the operation’s outcome, the duration of recovery to return to a daily routine and pre- and postoperative complaints respectively complications. The Austrian system of school marks was used to assess patient’s satisfaction ranging from 1 to 5; 1 equivalent to excellent and 5 equivalent to insufficient. The periods of recovery were classified into 5 periods (1: 1-3 days; 2: 4-7 days; 3: 8-14 days; 4: 15-21 days; 5:>21 days).

**Fig. 2:**
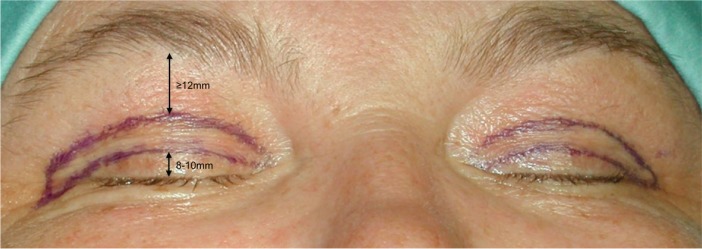
The lower incision line is placed in the supratarsal crease 8-10mm cephalad to the upper lid margin. After performing a pinch test the superior incision line is marked. The distance from the upper incision line to the lower brow margin should be at least 12mm

First, incision markings are drawn on bilateral upper lids along the supratarsal fold to outline inferior resection borders while having the patient in an upright sitting position. After performing a pinch test superior incision lines are then defined. We believe that the distance between superior incision lines and the lower border of the brow should be at least 12 mm. With typically lenticular medial margins and lateral triangular margins markings were completed (Figure 2). Both lids are then anesthetized with approximately 2 mL of 0.5% lidocaine with 1:100 000 fraction of epinephrine. 

**Fig. 3:**
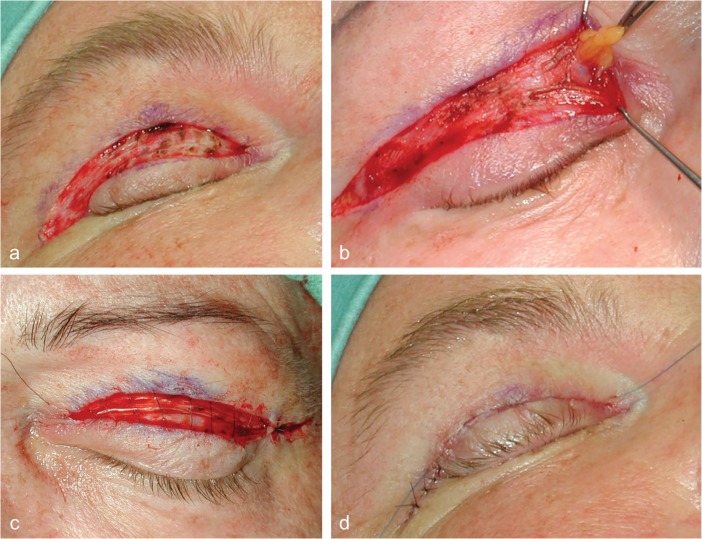
a) Intraoperative view after skin resection and hemostasis. b) Resection of fat from the nasal compartment following incision of the septum. c, d) Wound closure using running and interrupted simple sutures was then achieved

Next, skin incisions were carried out using a 15 blade scalpel. The skin flap was elevated and separated from the underlying tissue using small dissecting scissors or scalpel (Figure 3a). If muscle laxity was observed, an up to 5 mm measuring strip of orbicularis oculi muscle was then excised using dissecting scissors. In some cases, prolapsing fat tissue was present; resection was then carried out using either bipolar electrocautery or ligation and scissors (Figure 3b). Hemostasis was then accomplished using bipolar electrocoagulation. If needed laterally, a single deep dermal suture was placed (5-0 Vicryl). Skin closure was achieved using an intracutaneous 5-0 or 6-0 non-resorbable suture (Figure 3c, 3d).

The parameters overall satisfaction, period till return to public and return to work, patient’s aesthetic and scar-related ratings, complications, resected tissues (skin: S; skin+muscle: SM; skin+muscle+fat: SMF; skin+fat: SF) and medical preconditions and risk factors (Diabetes mellitus, arterial hypertension, oral anticoagulation and platelet aggregation inhibition therapy, nicotine consumption and other risk factors in particular chemotherapy, immune suppression, anti-estrogen treatment and coagulation disorders) were used for statistical analysis. 

Patients were grouped according to resected tissue (S, SM, SMF, SF). Satisfaction ratings and complication rates were compared using the Mann-Whitney U-test. For this purpose, prism 6® (GraphPad Software, Inc., La Jolla, CA, USA) and Microsoft Excel (Microsoft Corporation, 2011, Version 14.0) were used. Other relevant medical preconditions that could be considered risk factors were evaluated regarding satisfaction ratings and complications. Again, Mann-Whitney U – test was performed comparing the groups with different amounts of risk factors with the control group (no risk factors). *P *value<0,05 was considered statistically significant.

## RESULTS

In this study a retrospective analysis of 684 consecutive cases of upper lid blepharoplasty performed between January 2006 and June 2013 (range 6.5 years) was carried out by reviewing patient’s electronic health record. For a total of 387 cases, data collection was completed and therefore, included in this study. Most patients were female (n=323; 83.5%). Age ranged from 28 to 92 years with an average of 63.5 years old. Most patients underwent resection of skin, muscle and fat (n=166; 42.9%), in 24.6%, merely skin was removed. In 16.7%, skin and muscle and besides in 15.7%, skin and fat were excised. The Body Mass Index (BMI) averaged 26 kg/m^2^ (range=15.4-41.5). Ninety-five patients were smokers (24.5%). 

One hundred and twenty five patients presented with the risk factor hypertension. Nine out of 15 patients (60%), that had complications, had hypertension followed by patients that received anticoagulation or platelet antiaggregation therapy (5/15; 33.3%). Four out of 15 patients with complications were smokers (26.7%). Overall, 15 patients (3.9%) had complications. They have been classified into long-lasting (longer than 6 weeks) edema of the upper lid (5 patients; 33.3%), asymmetry (4 patients; 26.7%), wound dehiscence (3 patients; 20.0%), large excess of skin (2 patients, 13.3%) and epithelial cyst (1 patient; 6.7%). Revisional surgery has not been necessary in any cases.

Concerning overall satisfaction, 63.0% (244 patients) graded with 1 (excellent), 26.1% (101 patients) with 2 (good), 7.0% (27 patients) with 3 (satisfactory), 3.4% (13 patients) with 4 (sufficient) and 0.5% (2 patients) with 5 (insufficient). Totally, 364 patients (94.1%) would undergo surgery again. Easing of preoperative existing complaints after the operation was reported in 374 (96.6%) cases. Overall, 69.5% of all patients indicated that one week after surgery, they had already returned to a regular daily life and 58.4% had returned to work after one week, and 91.2% after two weeks. Assessing the aesthetic results, 250 patients (64.6%) rated with 1, 21.7% with 2, 10.3% with 3, 2.1% with 4 and 1.3% with 5. Scar assessment received slightly better marks, while 76.7% of patients rated with 1, 14.7% with 2, 5.2% with 3, 5.2% with 4 and 0.8% with 5.

Looking at patient’s overall outcome and assessing aesthetic and scar-related aspects we found no statistical significance between different kinds of tissue resection in our study (Table 1).

**Table 1:** Patients evaluation scores: tissue resections (S, SM, SF, SMF).

**Parameter**	**S (n=95)**	**SM (n=61)**		**SF (n=65)**		**SMF (n=166)**	
	**avg.±st.dev.**	**avg.±st.dev.**	***p*** ** value**	**avg.±st.dev.**	***p*** ** value**	**avg.±st.dev.**	***p*** ** value**
Overall satisfaction	1.63±0.89	1.46±0.77	0.27	1.58±0.92	0.70	1.46±0.73	0.17
Return to public	1.99±0.87	2.05±0.85	0.63	2.11±0.92	0.46	2.13±0.95	0.34
Return to work	2.29±0.97	2.21±0.95	0.69	2.28±0.96	0.97	2.39±0.94	0.36
Esthetics	1.60±0.88	1.52±0.92	0.47	1.62±0.96	0.94	1.46±0.77	0.22
Scar	1.34±0.75	1.49±0.92	0.10	1.32±0.69	0.97	1.40±0.84	0.67
Complications	2.08%	6.65%	0.21	4.62%	0.65	3.61%	0.71

An investigation of the different group’s average recovery periods (return to daily life/work) and complication rates, again, showed no significant difference between different types of tissue resections.

Comparing groups with different amounts of risk factors our study found three significant differences (Table 2). Only two patients with four risk factors have been included in our study. Compared to the control group (no risk factors) these patients had significantly longer recovery periods (Pat1: 8-14 d, Pat2: 15-21 d) till a return to public life was possible (0RF: avg=2,09; 4RF: avg=3,50; *p*=0,04). Besides, scar ratings showed to be significantly worse than in the control group (0RF: avg=1,37; 4RF: avg=3,00; *p*=0,01).

**Table 2:** Patient’s evaluation scores: no risk factors vs. risk factors.

**Parameter**	**Control / 0RF (n=183)**	**1RF (n=115)**		**2RF (n=70)**		**3RF (n=17)**		**4RF (n=2)**	
	**avg.±st.dev.**	**avg.±st.dev.**	***p*** ** value**	**avg.±st.dev.**	***p*** ** value**	**avg.±st.dev.**	***p*** ** value**	**avg.±st.dev.**	***p*** ** value**
Overall satisfaction	1.56±0.84	1.49±0.77	0.55	1.44±0.73	0.33	1.53±1.07	0.53	2.50±0.71	0.09
Return to public	2.09±0.87	1.97±0.88	0.22	2.23±1.05	0.47	1.82±0.73	0.26	3.50±0.71	0.04
Return to work	2.34±0.97	2.23±0.92	0.39	2.50±0.94	0.16	2.00±0.71	0.19	2.50±2.12	>0.99
Aesthetics	1.59±0.86	1.47±0.83	0.12	1.46±0.83	0.18	1.53±1.07	0.47	2.50±0.71	0.10
Scar	1.37±0.76	1.35±0.73	0.95	1.36±0.83	0.85	1.06±0.24	0.10	3.00±0	0.01
Complications	1.64%	2.61%	0.56	10.00%	0.02	11.76%	0.06	0%	>0,99

Moreover, comparison of complication rates showed significantly more events in the 2-risk factor group (0RF: avg=1,64%; 2RF: avg=10,00%; *p*=0,02) than in the control group. Similar correlations can be expected for the 3- and 4- risk factor groups; however, the group’s populations were too small to find subtle differences in these cases and hence no statistically significant difference was found. Nevertheless, it can be deduced that higher complication rates and longer recovery periods can be expected in patients with two risk factors or more.

## DISCUSSION

Upper lid blepharoplasty is generally considered a safe procedure with good outcomes and low complication rates.^12^^,^^13^ Nevertheless, various options of tissue resection exist. Even though consensus has not been reached yet on whether muscle is to be excised or not, traditionally, resection of the orbicularis oculi muscle was widely accepted.^14^^-^^17^ However, previous studies advocated sparing of the muscle for best preservation of a youthful fullness of the upper lid and to prevent a hollowed appearance of the periorbita.^18^^-^^20^

Contradicting this maxim Damasceno *et al.* believe that with respect to a youthful appearance not preservation of the muscle is most important but cautious resection of orbital fat.^12^ Moreover, further accentuation of the upper lid can be attained by rearranging resected fat from the medial compartment to enhance upper lid fullness.^18^^,^^19^^,^^21^ According to previous study, this can be feasibly accomplished by imbrication of the orbicularis oculi muscle.^18^ A similar technique was described before to improve lateral fullness of the upper lid by creating a double layer of orbicularis oculi muscle and inserting fat beads into this newly formed muscle pocket.^19^

Looking at patient’s overall outcome and assessing aesthetic and scar-related aspects we found no statistical significance between different kinds of tissue resection in our study (Table 1).

An investigation of the different group’s average recovery periods (return to daily life/work) and complication rates, again, showed no significant difference between different types of tissue resections. For this reason, we believe that cautious muscle stripping is merely indicated in cases of muscle laxity as our analysis shows equally good satisfaction ratings with no evidence of higher complication rates.

Comparing groups with different amounts of risk factors our study found three significant differences (Table 2). Only two patients with four risk factors have been included in our study. Compared to the control group (no risk factors) these patients had significantly longer recovery periods (Pat1: 8-14 d, Pat2: 15-21 d) till a return to public life was possible (0RF: avg=2,09; 4RF: avg=3,50; *p*=0,04). Besides, scar ratings showed to be significantly worse than in the control group (0RF: avg=1,37; 4RF: avg=3,00; *p*=0,01). 

Moreover, comparison of complication rates showed significantly more events in the 2-risk factor group (0RF: avg=1,64%; 2RF: avg=10,00%; *p*=0,02) than in the control group. Similar correlations can be expected for the 3- and 4- risk factor groups; however, the group’s populations were too small to find subtle differences in these cases and hence no statistically significant difference was found. Nevertheless, it can be deduced that higher complication rates and longer recovery periods can be expected in patients with two risk factors or more.

Even though no major complications that required revisional surgery occurred in our study, a fatal complication associated with blepharoplasty is the retrobulbar hematoma.^9^^,^^22^ Patients and surgeons have to be aware of imminent amaurosis in case of retrobulbar hemorrhage. Pain, amaurosis fugax, tense or expanding proptosis, scintillating scotoma and diplopic images are typical prodromes and due to a critical ischemia period of 90 to 120 min instant initiation of treatment is imperative to avert irreversible vision loss.^9^

To our best knowledge, this study is one of the largest evaluations of patient’s satisfaction that underwent functional blepharoplasty due to visual field restrictions. Overall, blepharoplasty is a common procedure in plastic surgery that basically has good results. However, there are still a few unanswered questions. Due to the fact that comparison of complication rates and patient’s satisfaction ratings regarding resected tissues revealed no statistically significant difference stripping of the orbicularis oculi muscle and/or resection of fat is merely advised if pathologies are present. 

Moreover, our study found that the presence of two risk factors or more accounts for a higher complication rate. In addition, patients with four risk factors had remarkably worse scar ratings and longer recovery periods till return to public life was possible. For this reason, indication for blepharoplasty in these cases is to be placed cautiously.
